# Effect of Hyperinsulinemia on Leptin and Ghrelin Levels in Polycystic Ovarian Syndrome: A Meta-Analysis

**DOI:** 10.7759/cureus.69023

**Published:** 2024-09-09

**Authors:** Mikyla Reesor, Yvette Goudiaby, Nicole Grossett, Natasha Zand, Royon Chichester, Luis Echevarria-Javier, Mykhailo Vysochyn, Amy Alam

**Affiliations:** 1 Clinical Sciences, Saint James School of Medicine, The Valley, AIA; 2 Cardiology, Saint James School of Medicine, The Valley, AIA

**Keywords:** ghrelin, hyperinsulinemia, insulin, leptin, meta-analysis, pcos, polycystic ovarian syndrome, reproductive

## Abstract

Polycystic ovarian syndrome (PCOS) is the most common endocrine disorder in women of reproductive age. Leptin and ghrelin are important markers in PCOS due to their correlation with obesity, insulin resistance, and fertility. There is currently a debate in the literature about whether altered leptin and ghrelin levels in women with PCOS are a result of the disease itself or if they are due to factors such as the hyperinsulinemic state characteristic of PCOS. This meta-analysis aims to assess if insulin levels impact leptin and ghrelin levels in PCOS.

Eight case-control studies assessing the relationship between insulin and leptin, as well as five case-control studies assessing the relationship between insulin and ghrelin, were identified in PubMed. Pearson’s correlation coefficient (PCC) and the sample size were extracted, and two meta-analyses were conducted using a random-effects model. Total heterogeneity (I2) with a confidence interval of 95% was then determined. “Leave-one out” diagnostics were calculated for each case. If a study was identified as being significantly influential, the study was removed from the data set, and the trim and fill procedure was applied. Publication bias was assessed using Egger’s regression test and rank correlation test.

Our results showed a moderate positive relationship (r=0.56, 95% confidence interval (CI) (0.42, 0.71), with substantial heterogeneity I^2^=81.35%, 95% CI (25.2799, 88.2451)) between insulin and leptin levels, and a moderate negative relationship (r=-0.33, 95% CI (-0.43, -0.24)), with low heterogeneity (I^2^=0.00%, 95% CI (0.0000, 80.8159)) between insulin and ghrelin levels.

Therefore, there is a significant relationship between insulin and higher leptin and lower ghrelin levels in women with PCOS. Better insulin control may have a positive effect on fertility, appetite, weight, body image, and quality of life in these women. This correlation is likely multifactorial, and further studies are needed to isolate factors influencing these hormones.

## Introduction and background

Polycystic ovarian syndrome (PCOS) is the most common endocrine disorder in women of reproductive age, affecting 5-10% of women [1]. According to the Rotterdam criteria, PCOS is characterized by at least two of the following abnormalities: oligo- and/or anovulation, clinical and/or biological hyperandrogenism, and polycystic ovaries diagnosed on transvaginal ultrasound [2]. The concern with this syndrome lies in the significance of associated complications. Women with PCOS experience reproductive complications such as menstrual dysfunction, infertility, and pregnancy complications [1]. Insulin resistance is the most common metabolic feature, found in almost 35-80% of women with PCOS, and is independent of body mass index (BMI) and body fat distribution [1]. PCOS is a well-known state of chronic hyperinsulinemia as a result of attempting to compensate for insulin resistance [3]. PCOS is highly comorbid with type 2 diabetes mellitus and cardiovascular disease (CVD) as a result of this state [1]. 

Studies have previously been conducted noting the relationship between insulin with leptin and ghrelin in PCOS. Leptin is a peptide hormone released by adipocytes. It acts by inhibiting orexigenic (appetite-stimulating) neurons and activating anorexigenic (appetite-suppressing) neurons in the hypothalamus to suppress appetite [4]. Insulin has been shown to induce leptin gene expression, suggesting that insulin may stimulate leptin secretion [5]. Ghrelin is a multifunctional peptide hormone that induces increased appetite, glucose release, cell proliferation, and reproduction [6]. Leptin and ghrelin are not only significant due to their influence on satiety and hunger in the hypothalamus, but these hormones also influence the secretion of gonadotropin-releasing hormones and thus impact fertility [7]. 

Many studies have reported elevated leptin levels in women with PCOS, while other studies have found no difference in leptin levels between women with PCOS and those without. The latter studies suggest that leptin levels may be influenced more by individual factors such as insulin levels, age, anthropometrics, and genetics rather than being a characteristic of PCOS itself [8]. On the other hand, many studies have shown there to be lower ghrelin levels in women with PCOS, proposing that lower levels are due to higher body mass indexes (BMI), while other studies have demonstrated no significant change in ghrelin levels in PCOS [8]. 

This study will comprehensively analyze the relationship between insulin with leptin and ghrelin in reproductive-aged women with PCOS in case-control studies assessing this relationship.

This article was previously presented as a poster at the 28th Annual Poster Exhibition at the American Medical School Association’s Future Physicians for Change Conference on June 17, 2023.

## Review

Methods

Literature Search

**Figure 1:**
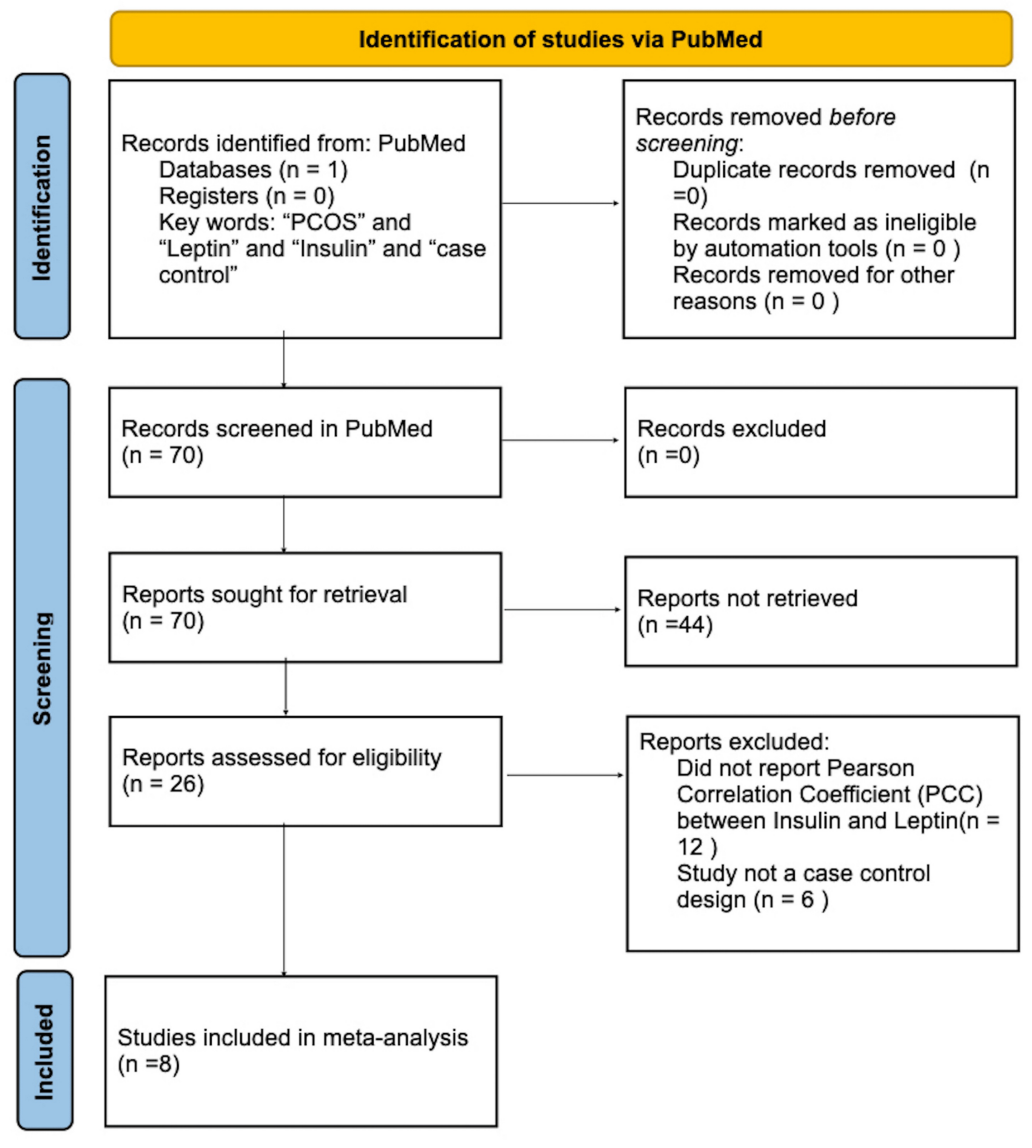
PRISMA flow chart of the literature search for case-control studies assessing the correlation between insulin and leptin in women with PCOS. PRISMA: Preferred Reporting Items for Systematic Reviews and Meta-Analyses; PCOS: polycystic ovarian syndrome

**Figure 2:**
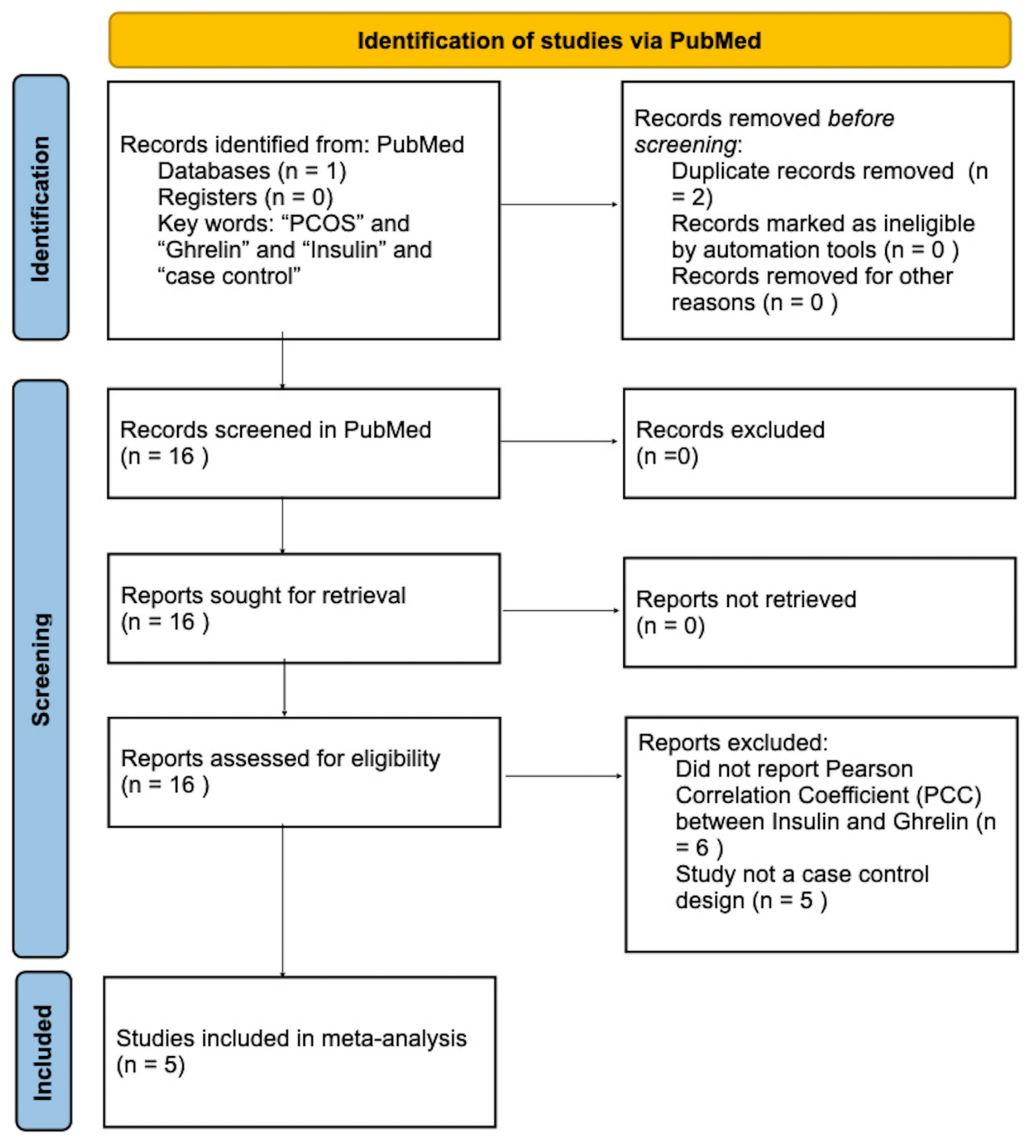
PRISMA flow chart of the literature search for case-control studies assessing the correlation between insulin and ghrelin in women with PCOS. PRISMA: Preferred Reporting Items for Systematic Reviews and Meta-Analyses; PCOS: polycystic ovarian syndrome

Relevant literature was searched in PubMed from June 1, 2022, to November 5, 2022, for case-control studies published in English. Two searches were completed to identify studies assessing the correlation between insulin and leptin (Figure 1) and insulin in ghrelin (Figure 2) in women with PCOS.

*Selection Criteria* 

Studies that were included were papers that defined PCOS according to the Rotterdam criteria; used a case-control study design that compared women with PCOS to that of healthy matched controls; and reported the relationship between insulin and leptin/ghrelin using Pearson’s correlation coefficient (PCC). Additionally, studies were excluded if they were reviews or editorials; they included post-menopausal women or pregnant women. 

Data Extraction

The following items were extracted from each relevant study: first author, sample number, average insulin levels, average leptin levels, average ghrelin levels, Pearson’s correlation coefficient between; insulin and leptin, and/or insulin and ghrelin. 

Statistical Analysis

The Pearson correlation coefficient (PCC) examining the relationship between insulin and leptin and/or insulin and ghrelin was extracted. R Studio version 4.2.2 was used to perform the following analysis. The first PCC was transformed to Fisher’s Z to calculate the corresponding sample variances. The meta-analysis was then performed using a random effects model to account for heterogeneity in the included studies.

The degree of heterogeneity (tau^2^) was calculated using a restricted maximum-likelihood estimator. Total heterogeneity (I^2^) with a confidence interval of 95% was then determined, as well as the Q-statistic with degrees of freedom and p-value. The estimated model coefficient, or summary effect size, of each study and the standard error were plotted using a Baujau plot to illustrate studies influencing overall heterogeneity. To further identify potential outliers and influential cases, the following “leave-one-out” diagnostics were calculated for each case: externally standardized residuals, DFFITs value, Cook’s distances, covariance ratio, the “leave-one-out” amount of residual heterogeneity, the “leave-one-out” test statistic for the test of residual heterogeneity, and weight. If a study was identified as being significantly influential, the study was removed from the data set, and the trim and fill procedure was applied. 

Publication bias was assessed using Egger’s regression test and rank correlation test. A funnel plot of the biased data set and a forest plot with 95% confidence intervals (CI) were also produced using this interface. 

Results

Correlation of Insulin and Leptin in PCOS

**Figure 3:**
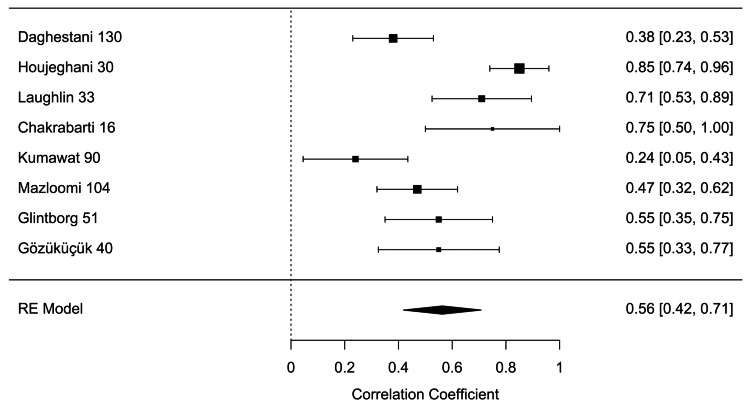
Forest plot of random effects estimates with 95% confidence intervals. Tau^2^=0.0791 (SE=0.0550), tau=0.2812, I^2^=81.35%, H^2^=5.36, Q(df=7)=30.7460, p<0.0001 [8-15]

**Figure 4:**
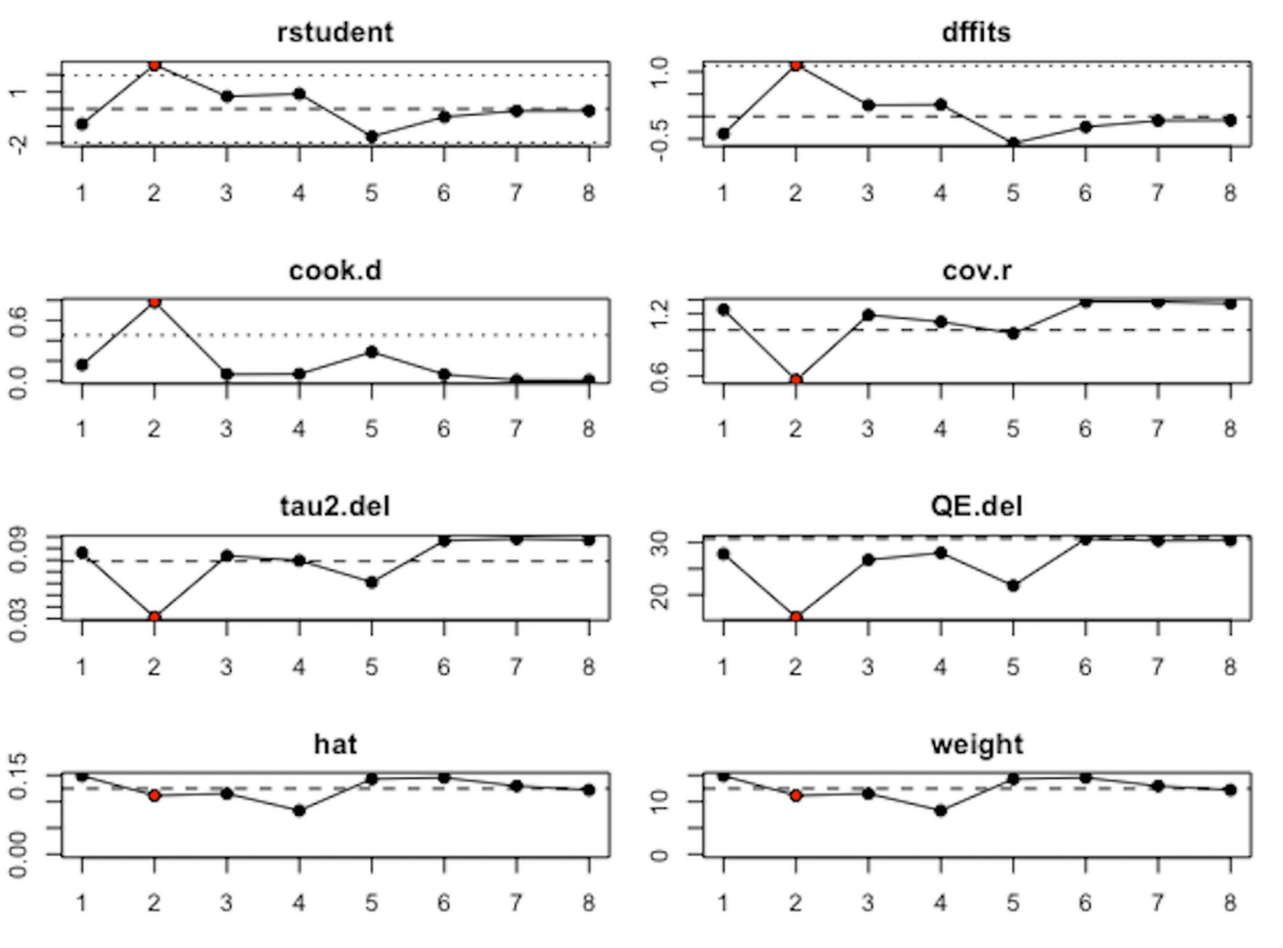
Influence of case analysis demonstrating study 2 to be an outlier. Influence of case analysis tests: externally standardized residuals, DFFITs value, Cook's distances, covariance ratio, the "leave-one-out" amount of residual heterogeneity, the "leave-one-out" test statistic for the test of residual heterogeneity, and weight.

**Figure 5:**
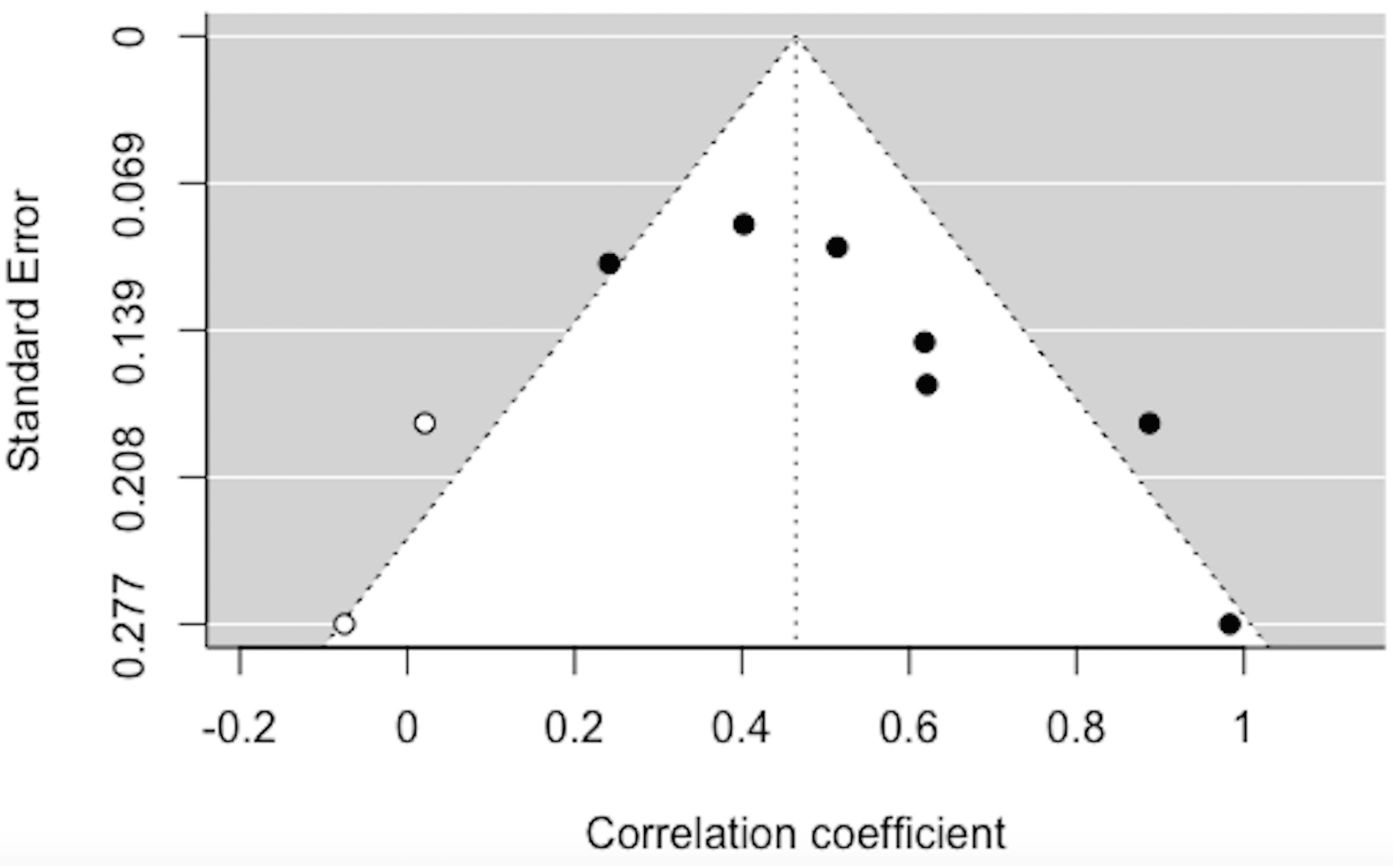
Funnel plot adjusted using the trim-and-fill method. white circles: comparisons included; black circles: inputted comparisons using the trim-and-fill method

Eight of the included studies reported a Pearson correlation coefficient (PCC) of insulin and leptin in women with PCOS between 0.38 and 0.85 (p<0.01) (Table 1). The results of the meta-analysis of the eight studies with a randomized-effect model showed a moderate positive relationship (r=0.56, 95% CI (0.42, 0.71), with substantial heterogeneity I^2^=81.35%, 95% CI (25.2799, 88.2451)) between insulin and leptin levels in women with PCOS (Figure 3). Egger’s regression test (p=0.0003) and rank correlation test (p=0.0312) were significant for funnel plot asymmetry, showing that there may have been evidence of publication bias according to these tests. The study by Houjeghani et al. was identified as a potential outlier due to the small effect size and high standard error, so the trim and fill procedure was done to remove this study as a source of influence and bias to the results (Figures 4, 5) [9].

**Table 1:** Studies included in the meta-analysis with the sample sizes of women in the PCOS group and control group and PCC of insulin and leptin. PCC: Pearson correlation coefficient; PCOS: polycystic ovarian syndrome [8-15]

Study First Author Last Name	Sample Size PCOS	Sample Size Controls	PCC PCOS Insulin Leptin
Daghestani	130	122	0.382
Houjeghani	30	30	0.85
Laughlin	33	32	0.71
Chakrabarti	16	18	0.75
Kumawat	90	90	0.24
Mazloomi	104	99	0.47
Glintborg	51	63	0.55
Gözüküçük	40	40	0.55

*Correlation of Insulin and Ghrelin in PCOS* 

**Figure 6:**
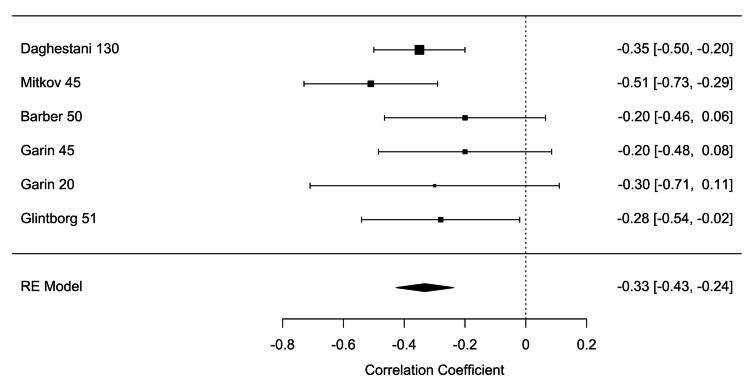
Forest plot of random effects estimates with 95% confidence intervals. Tau^2^=0.0311 (SE=0.0294), tau=0.1764, I^2^=64.85%, H^2^=2.84, Q(df=6)=15.7218, p=0.0153 [8,10,16-18]

**Figure 7:**
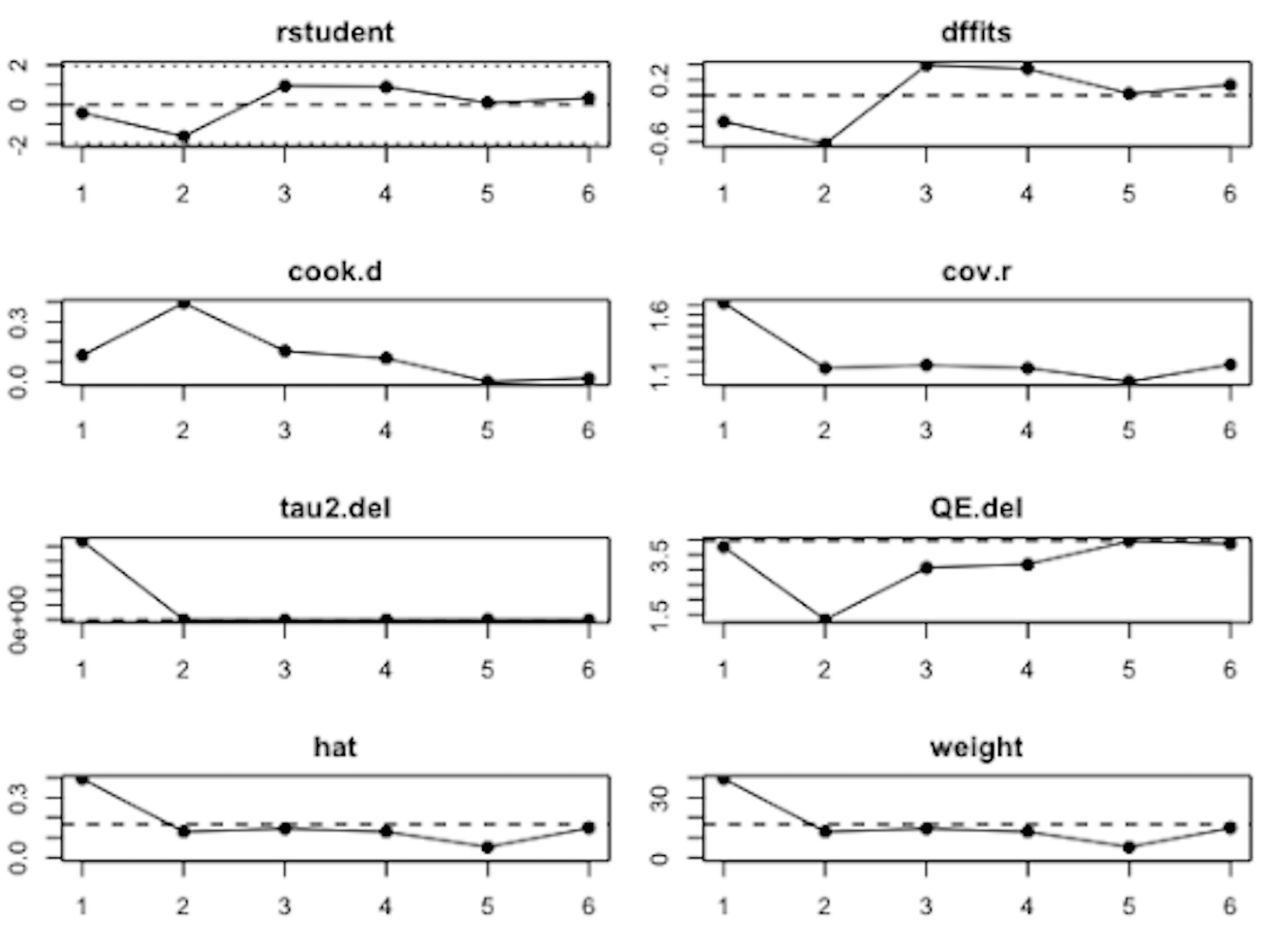
Influence of case analysis demonstrating no potential outliers. Influence of case analysis tests: externally standardized residuals, DFFITs value, Cook's distances, covariance ratio, the "leave-one-out" amount of residual heterogeneity, the "leave-one-out" test statistic for the test of residual heterogeneity, and weight.

**Figure 8:**
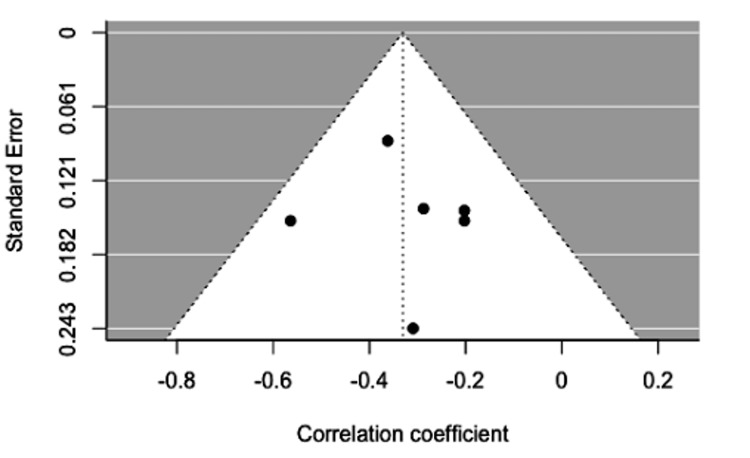
Funnel plot without adjustment for publication bias.

Five of the included studies reported a Pearson correlation coefficient (PCC) of insulin and ghrelin in women with PCOS between -0.51 and -0.2 (p<0.05) (Table 2) (Figure 6). The study by Garin et al. reported two PCCs that assessed the relationship between women categorized as obese and another between women categorized as lean, both of which were included in the meta-analysis [16]. The results of the meta-analysis of the five studies with a randomized-effect model showed a moderate negative relationship (r=-0.33, 95% CI (-0.43, -0.24)), with low heterogeneity (I^2^=0.00%, 95% CI (0.0000, 80.8159)) between insulin and ghrelin levels in women with PCOS. Egger’s regression test (p=0.7541) and rank correlation test (p=1.000) were insignificant for funnel plot asymmetry, showing that there was no evidence of publication bias according to these tests (Figure 7). No potential outliers were identified, so the trim and fill procedure was not applied (Figure 8). 

**Table 2:** Studies included in the meta-analysis with the sample sizes of women in the PCOS group and control group and PCC of insulin and ghrelin. PCC: Pearson correlation coefficient; PCOS: polycystic ovarian syndrome [8,10,16-18]

Study First Author Last Name	Sample Size PCOS	Sample Size Controls	PCC PCOS Insulin Ghrelin
Daghestani	130	122	-0.35
Mitkov	45	20	-0.51
Barber	50	28	-0.2
Garin	45	33	-0.2
Garin	20	21	-0.3
Glintborg	51	63	-0.28

Discussion

Leptin and ghrelin have been indicated as important markers in polycystic ovarian syndrome (PCOS) due to their correlation with obesity, insulin resistance, and fertility [1]. There is currently a debate in the literature about whether altered leptin and ghrelin levels in women with PCOS are a result of the disease itself or if they are due to other contributing factors such as BMI or insulin resistance [1]. The purpose of this study is to establish if the hyperinsulinemic state characterizing PCOS is a significant contributing factor to leptin and ghrelin hormones. Establishing this correlation can provide physicians with insight into factors influencing eating habits, anthropometrics, and fertility challenges within this population. 

Many studies have reported that women with PCOS have a higher risk of developing eating disorders and have found that women with PCOS have higher levels of leptin and lower levels of ghrelin compared to their healthy matched controls [1,8,9,17,19]. Suppression of appetite would therefore be the result of these altered hormone levels. Obesity is one of the comorbidities of PCOS, and patients are often advised by their physicians to lose weight in an attempt to improve their symptoms [20]. It’s important for physicians to be conscientious of both the possibility of inherent hormonal appetite reduction and the increased risk of eating disorders in this population when recommending lifestyle modifications to improve insulin resistance. Recommending calorie restriction as a means of weight loss to improve symptoms may be counterproductive if the woman’s appetite is already suppressed by this hormonal imbalance. 

Infertility is one of the greatest challenges that women with PCOS experience [7]. Though the exact pathophysiology of PCOS remains unknown, the hypothalamic-pituitary-gonadal axis disturbances in PCOS are believed to be caused by insulin resistance that ensues either from genetic predisposition or lifestyle [11]. Insulin resistance, and the consequent hyperinsulinemia, stimulate androgen production in ovarian theca cells and reduce sex hormone-binding globulin (SHBG) production in the liver. Elevated free androgen levels then trigger excessive luteinizing hormone (LH) secretion from the anterior pituitary gland. The elevated LH levels relative to follicle-stimulating hormone (FSH) levels impair follicular development, leading to anovulation. Increased pulsatility of gonadotropin-releasing hormone (GnRH) in the hypothalamus and peripheral conversion of androgens to estrogen in adipose tissue further exacerbate the LH/FSH imbalance [1,10,11]. 

While normal levels of leptin induce sex steroid synthesis and oocyte development in the ovaries, excessively high levels of leptin have demonstrated a decrease in the response of the ovaries to gonadotropins and subsequently have been shown to lead to ovarian cyst development [7]. Insulin ensures successful ovulation through the regulation of gonadotropin hormone receptors and GnRH pulse frequency in the ovaries [7]. The presence of insulin resistance may also limit leptin’s ability to induce hormone synthesis and oocyte development in the ovaries. Leptin acts centrally to stimulate the secretion of gonadotropin-releasing hormone from the hypothalamus and thus the secretion of luteinizing hormone (LH) and follicle-stimulating hormone (FSH) [7]. Adipose tissue is the primary site of leptin synthesis; however, it is also synthesized in granulosa cells in the ovary. Leptin also regulates insulin secretion from pancreatic beta cells and insulin’s actions on adipocytes and skeletal muscle [1]. The presence of central leptin resistance due to chronic inflammation in PCOS cases also prevents leptin from fully performing its physiological functions in the hypothalamic-pituitary-ovarian axis and negatively affects fertility [7]. 

Many studies have reported a significant positive correlation between insulin and leptin in PCOS, while other studies have noted this relationship to be insignificant. Yurci et al. found that serum leptin showed a positive correlation with insulin (r=0.74, p<0.03) [7]. This study, however, was analyzing women with PCOS who were also clomiphene-resistant. A study by Nasrat et al. demonstrated that leptin positively correlated with insulin resistance, but the correlation was not significant (r=0.148, p=0.258) [3]. An insignificant correlation was also reported by Mancini et al. [21]. Erturk et al. reporting (r=0.317, p<0.5) results demonstrated that hyperleptinemia in women with PCOS is correlated only with obesity and is not affected by insulin levels [22]. The study by Cohen et al. showed that leptin inhibits insulin binding in adipocytes and attenuates several insulin-induced actions in hepatocytes [4]. They concluded that high serum leptin levels are related to obesity, hyperinsulinemia, and insulin resistance [4,23]. Daghestani et al. found through multiple regression analysis that insulin is the primary determinant of leptin levels in women with PCOS (r2=0.352, p<0.0001) [8]. 

Our meta-analysis demonstrated a moderately significant positive relationship (r=0.56, 95% CI (0.42, 0.71)) between insulin and leptin levels in women with PCOS. Substantial heterogeneity (I^2^=81.35%, 95% CI (25.2799, 88.2451)) was observed, however, limiting the generalizability of our results. The study by Houjeghani et al. was identified as an outlier influencing the data set [9]. This study had a relatively low sample size, a narrow range of BMI, and excluded women who had other concurrent medical illnesses, metabolic syndromes, and those who were on oral contraceptives, glucocorticoids, anti-androgens, ovulation induction agents, antidiabetic and anti-obesity drugs, or other hormonal drugs. This may indicate that the study population either experienced milder symptoms of PCOS that did not require treatment or that their symptoms were not previously treated or controlled [9].

While leptin induces the secretion of gonadotropin-releasing hormone, ghrelin decreases the secretion of gonadotropin-releasing hormone from the hypothalamus [7]. Ghrelin also regulates glucose homeostasis through inhibition of insulin secretion and regulation of hepatic glucose output [24]. Low ghrelin levels have been associated with higher BMI, insulin resistance, and diabetes [25]. While it is established that PCOS can be related to obesity and insulin resistance, ghrelin’s role in the pathophysiology of PCOS remains undetermined. A meta-analysis including 894 PCOS patients and 574 controls revealed that ghrelin levels were significantly lower in PCOS patients than in controls, with a standardized mean difference of −0.40 (95% CI (−0.73, −0.08) [26].

Many studies have reported a significant negative correlation between insulin and ghrelin in PCOS, while other studies have noted this relationship to be insignificant. Glintborg et al. initially found a significant negative correlation between insulin and ghrelin in PCOS patients and controls; however, after adjusting for fat mass, they reported this relationship to be insignificant [10]. Temel et al. reported no significant correlation between fasting insulin and ghrelin; however, they did report a positive correlation between ghrelin levels and the Homeostatic Model Assessment of Insulin Resistance (HOMA-IR) [27]. Schöfl et al. found that in insulin-sensitive PCOS cases, ghrelin concentrations compared well with the healthy controls, whereas in insulin-resistant PCOS cases, ghrelin concentrations were significantly lower and indistinguishable from the low levels found in the gastrectomized women [28]. Mitkov et al. found that serum ghrelin levels were significantly decreased in women with PCOS compared to their healthy matched controls, and the difference persisted after controlling for age, BMI, waist-hip-ratio (WHR), and waist circumference (WC) [17]. A strong negative correlation was initially observed between ghrelin values and insulin and HOMA-IR in women with PCOS; however, the relationship diminished after partial correlation analysis when controlling for WHR, BMI, and WC. To further establish the effect of HOMA-IR on ghrelin values, the women with PCOS were divided into two groups: with HOMA-IR ≤ 2.5 (group A, n=22) and with HOMA-IR ﹥ 2.5 (group B, n=23). In group B with more prominent insulin resistance, ghrelin levels were significantly lower (29.04 ± 3.42 vs. 18.64 ± 2.50 ng/ml; p=0.09). A negative correlation between serum ghrelin and leptin levels was also determined; however, the correlation was not significant after controlling for metabolic parameters [17]. This study may have been limited by the fact that total ghrelin levels were included. Inactive plasma ghrelin accounts for over 90% of circulating ghrelin, and the ratio of inactive to active ghrelin can vary [17]. 

Our meta-analysis demonstrated a moderate negative relationship (r=-0.33, 95% CI (-0.43, -0.24)), with low heterogeneity I^2^=0.00%, 95% CI (0.0000, 80.8159) between insulin and ghrelin levels in women with PCOS. The calculated confidence interval did not contain the value of zero; therefore, we were able to reject the null hypothesis and state that the relationship is significant. Therefore, higher insulin levels in women with PCOS correspond to lower levels of the appetite-stimulating hormone ghrelin. 

Limitations 

There was no subgroup analysis performed for moderators that may be influencing the results of our meta-analysis, such as BMI, age, HOMA-IR, and the level of the quality of evidence of each study. Additionally, the quality of evidence in each study was not objectively graded using a systematic method. 

## Conclusions

There is a significant relationship between insulin levels and leptin and ghrelin in women with PCOS, with higher insulin levels corresponding to higher leptin and lower ghrelin levels. It is therefore important for physicians to consider that insulin resistance in PCOS may correspond with appetite suppression. Recommending calorie restriction as a means of weight loss to improve insulin resistance and PCOS symptoms may be counterproductive to the goals of the patient if they are already having difficulty eating. If insulin levels can therefore be controlled, this may have positive effects on fertility, appetite, weight control, body image, and quality of life.

This correlation is likely multifactorial, however, and further studies are needed to isolate factors influencing these hormones. 
